# Detection of circulating vaccine-derived poliovirus type 2 (cVDPV2) in wastewater samples: a wake-up call, Finland, Germany, Poland, Spain, the United Kingdom, 2024

**DOI:** 10.2807/1560-7917.ES.2025.30.3.2500037

**Published:** 2025-01-23

**Authors:** Sindy Böttcher, Julian Kreibich, Thomas Wilton, Vanessa Saliba, Soile Blomqvist, Haider Al-Hello, Carita Savolainen-Kopra, Magdalena Wieczorek, Beata Gad, Arleta Krzysztoszek, Rosa M Pintó, María Cabrerizo, Albert Bosch, Eugene Saxentoff, Sabine Diedrich, Javier Martin

**Affiliations:** 1 Robert Koch Institute (RKI), Berlin, Germany; 2 Medicines and Healthcare products Regulatory Agency (MHRA), South Mimms (Potters Bar), United Kingdom; 3 United Kingdom Health Security Agency (UKHSA), London, United Kingdom; 4 Finnish Institute for Health and Welfare (THL), Helsinki, Finland; 5 National Institute of Public Health NIH – National Institute of Research, Warsaw, Poland; 6 University of Barcelona, Barcelona, Spain; 7 Instituto de Salud Carlos III (ISCII), Madrid, Spain; 8 World Health Organization Regional Office for Europe (WHO/Europe), Copenhagen, Denmark

**Keywords:** poliovirus, poliomyelitis, VDPV, wastewater, PHEIC, eradication

## Abstract

In 2024, circulating vaccine-derived poliovirus type 2 (cVDPV2) was detected in wastewater samples in Finland, Germany, Poland, Spain and the United Kingdom (UK). All strains were genetically linked, but sequence analysis showed high genetic diversity among the strains identified within individual wastewater sites and countries and an unexpected high genetic proximity among isolates from different countries. Taken together these results, with sequential samples having tested positive in various sites, a broader geographic distribution beyond positive sampling sites must be considered.

Since the Global Polio Eradication Initiative (GPEI) was founded in 1988 [1], great progress has been made towards a poliovirus-free world. Nevertheless, the risk of international spread still exists due to the continued emergence of vaccine-derived poliovirus (VDPV) outbreaks and the continued circulation of wild-type 1 poliovirus in areas of Afghanistan and Pakistan. Therefore, a Public Health Emergency of International Concern (PHEIC) was announced in 2014 and is still in place [2].

Since September 2024, circulating VDPV type 2 (cVDPV2) has been detected in wastewater samples within routine and research environmental surveillance (ES) activities in five European countries: Finland, Germany, Poland, Spain and the United Kingdom (UK). Here we describe the surveillance, detection and characterisation of cVDPV2.

## Environmental surveillance for poliovirus in five European countries

To survey the circulation of polioviruses, countries are responsible of maintaining appropriate surveillance systems. As a supplementary system to clinical diagnosis, wastewater-based poliovirus surveillance offers the opportunity to detect the reintroduction or circulation of polioviruses in the population at an early stage and to take appropriate measures to minimise their spread.

Environmental surveillance for poliovirus is conducted within the framework of routine programmes (Finland, Poland, UK) or research projects (Germany, Spain) to support the GPEI. The reporting laboratories are accredited by the World Health Organization (WHO) Global Polio Laboratory Network (GPLN) and represent either national (Poland, Spain), regional (Finland, Germany) or global (UK) specialised reference laboratories for poliovirus surveillance. Analysis workflows are based on WHO recommendations [3] and include virus isolation by cell culture and/or molecular methods (PCR, sequencing); protocols are published or available upon request. Details of the methods used in the different laboratories and dates of the first cVDPV2 detections in wastewater are shown in Table.

**Table:** Overview of sampling sites and methods used routinely for detection and characterisation of poliovirus from wastewater samples, Finland, Germany, Poland, Spain, the United Kingdom, 2024

Country	Sampling sites (n)	Testing frequency	Wastewater processing protocol and enterovirus including poliovirus detection	Molecular characterisation	Whole genome analysis	First detection of cVDPV2	
						Date	Sampling sites (n)
Finland	5	Biweekly (n = 1) and monthly (n = 4)	2-phase PEG/dextran separation, virus isolation in cell culture [3]	ITD [5], VP1 amplification and Sanger sequencing [6]	RNA extraction, (near) full genome RT-PCR and ONT sequencing	18 Nov	1
Germany	7	Weekly (n = 3) and monthly (n = 4)	PEG/NaCl precipitation and virus isolation in cell culture [3]	VP1 amplification and Sanger sequencing [6]	RNA extraction, cDNA synthesis and Illumina sequencing	28 Oct	7
Poland	8	Weekly (n = 2) and biweekly (n = 6)	SiO_2_ protocol [20] and virus isolation in cell culture [3]	ITD [5], VP1 amplification and Sanger sequencing by RKI [6]	RNA extraction, (near) full genome RT-PCR and ONT sequencing by MHRA [7]	22 Oct	2
Spain	2	Biweekly	Al(OH)3 precipitation [21] and direct detection	VP1 amplification and ONT sequencing [8]	ND	16 Sep	1
United Kingdom	12 plus 14 sampling sites tested by direct detection method only	Monthly and biweekly when cVDPV2 found	Size exclusion filter centrifugation, virus isolation in cell culture and direct detection by ONT sequencing DDNS [7]	ITD [5], VP1 amplification and ONT sequencing [7]	RNA extraction, (near) full genome RT-PCR and ONT sequencing [7]	5 Nov	4

cDNA: complementary DNA; cVDPD2: circulating vaccine-derived poliovirus type 2; DDNS: direct detection by nanopore sequencing; ITD: intratrypic differentiation; MHRA: Medicines and Healthcare products Regulatory Agency, the United Kingdom; ND: not done; ONT: Oxford nanopore technology; PEG: polyethylene glycol; RKI: Robert Koch Institute, Germany; RT-PCR: reverse-transcription PCR; VP1:  virus protein 1.

All countries responded to the poliovirus detections following WHO recommendations [4]. All detections were reported via International Health Regulations (IHR; https://www.who.int/) and Early Warning and Response System (EWRS; https://ewrs.ecdc.europa.eu/) so that a quick response including risk assessment could be carried out. Timely collaboration organised by the WHO Regional Office for Europe (WHO/Europe) involving the WHO headquarters, the United States (US) Centers for Disease Control and Prevention (CDC) and European Centre for Disease Prevention and Control (ECDC) enabled quick data exchange and analysis of sequencing results.

### Handling of wastewater samples for poliovirus testing

All activities involving wastewater were conducted in separate laboratory areas to avoid simultaneous processing of clinical samples and wastewater samples. All laboratories work under appropriate biocontainment requirements.

### Cell culture isolation and molecular characterisation of polioviruses

Two different cell lines were used: a transgenic mouse cell line, highly specific to poliovirus infection due to carrying the human poliovirus receptor (L20B) and a human cell line (RD-A), very sensitive for infection by polio and non-polio enteroviruses (NPEV), providing the best combination to increase the chance for poliovirus detection. Detection of cytopathic effect (CPE) on RD-A cells, expected from most wastewater samples due to the ubiquitous presence of NPEV, also serves as a control to confirm that processing of wastewater samples was correct. Virus isolation on L20B cells either directly or through cross passaging between RD-A and L20B cells allows isolation of poliovirus. Cultures of L20B with CPE are selected for further molecular characterisation by intratypic differentiation (ITD) (CDC, catalogue number GR-139) [5] and/or by amplification of the virus protein 1 (VP1) region [6] and/or the near whole genome region [7] by reverse transcription (RT)-PCR followed by Sanger or Oxford nanopore technology (ONT) sequencing.

### Direct detection of polio and non-polio enteroviruses

Direct detection of enteroviruses from concentrated wastewater samples was either performed by partial or full VP1 amplification followed by ONT sequencing using published protocols [7,8].

### Nucleotide sequence analysis

Sequencing data were processed and analysed using Geneious Prime version 2020.0.3 (https://www.geneious.com/), Sequencher 5.4.6 (https://www.genecodes.com/) and MEGA X (https://www.megasoftware.net/). The VP1 sequences (903 nt) generated from PCR products from virus isolates or directly from wastewater concentrates were aligned and compared with the sequence of the Sabin 2 vaccine reference strain (GenBank (https://www.ncbi.nlm.nih.gov/genbank/) identification code (ID): AY184220). The VP1 phylogenetic relationships between all cVDPV2 isolates were inferred using the maximum likelihood method and Tamura–Nei model within Geneious Prime version 2020.0.3 software and the phylogenetic tree was visualised using Microreact software [9]. Whole genome sequences from virus isolates generated by ONT sequencing were analysed by alignment to the Sabin 2 vaccine reference sequence to identify possible recombination events revealed by differences in the degree of sequence identity to the vaccine strain across the genome.

### Detection of circulating vaccine-derived poliovirus type 2 (cVDPV2)

**Figure 1:**
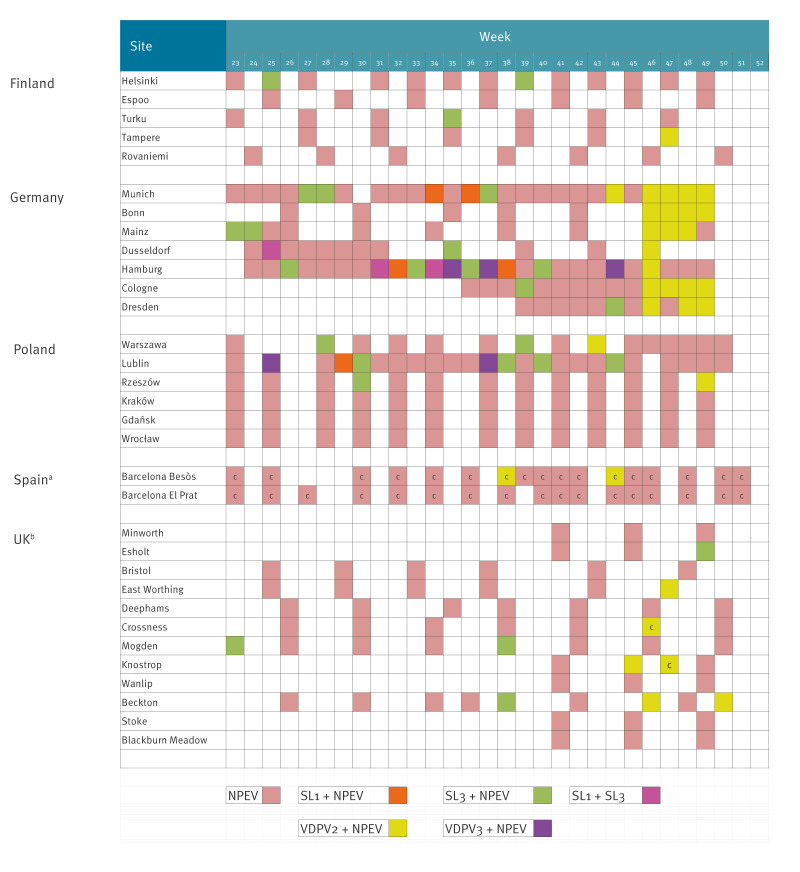
Overview on wastewater sampling sites, routine schedule and samples tested for poliovirus, Finland, Germany, Poland, Spain, the United Kingdom, September–December 2024 (n = 337)

Results of poliovirus detections from wastewater samples from the five countries involved are shown in Figure 1. Besides NPEV strains, sporadic detections of oral polio vaccine (OPV) strains (Sabin-like (SL)) type 1 and 3 and VDPV strains type 3 (VDPV3) were identified during the period reported, proving the high sensitivity of the system. Since the OPV is not used in the countries reporting here, these virus strains are most probably shed by individuals travelling from countries using bivalent OPV (types 1 and 3) in their vaccination schedule. Circulating VDPV2 strains were detected in five countries with different frequency, with the virus found in Finland in only one sample and in Spain and Poland in only two samples. In Germany, cVDPV2 strains were detected in wastewater samples in all sites sampled, and in some sites, the virus was found for four continuous weeks. In the UK, cVDPV2 strains were found in various sites but at a lower frequency than in Germany. These differences in cVDPV2 detections suggest different levels of infection in the five countries but might also reveal differences in the sensitivity for poliovirus detection from the different wastewater sites.

cVDPV2: circulating vaccine-derived poliovirus type 2; NPEV: non-polio enteroviruses; SL: Sabin-like strain; VDPV: vaccine-derived poliovirus; VP: virus protein.

^a^ cVDPV2 was detected only by direct detection in Spain, a 2,900 nt sequence covering the entire capsid region was obtained from the first positive sample while only a 331 nt VP1 fragment was recovered from the second positive sample.

^b^ 14 additional sites are monitored by direct detection only, all negative for cVDPV2 during this period.

^c^ Direct detection.

Of the 337 samples tested, 40 were from Finland, 110 from Germany, 91 from Poland, 34 from Spain and 62 from the United Kingdom.

### Genetic characterisation of circulating vaccine-derived poliovirus type 2 (cVDPV2) strains

All cVDPV2 strains were genetically related to each other and showed 43–52 VP1 nt differences from the Sabin 2 vaccine reference strain. Initial analysis conducted at CDC identified all European cVDPV2 strains as genetically linked to cVDPV2 emergence NIE-ZAS-1 [10], first detected in June 2020 in Zamfara, Nigeria, and actively circulating in various African countries in recent years [11]. Classification of cVDPV2 met the GPEI requirements for VDPV classification and reporting (https://polioeradication.org/wp-content/uploads/2016/09/Reporting-and-Classification-of-VDPVs_Aug2016_EN.pdf). The European cVDPV2 strains form a separate cluster within NIE-ZAS-1 branching pattern and showed 13 VP1 nt differences from the parent cluster suggesting at least 1 year of undetected circulation leading to these detection events, likely occurring outside of the catchment areas of these European ES sites, as otherwise viruses would have been detected earlier [10].

**Figure 2:**
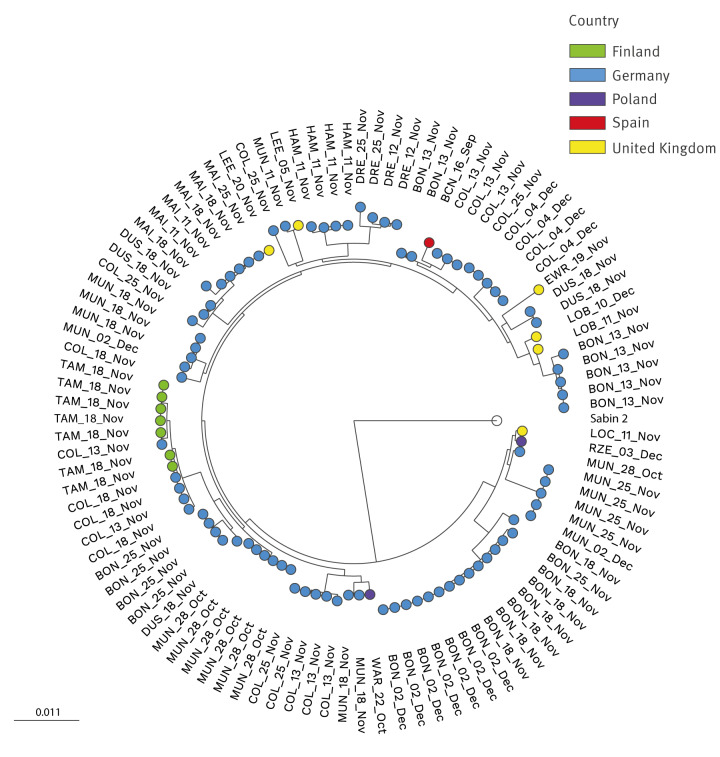
Phylogenetic analysis using the virus protein 1 (VP1) region of circulating vaccine-derived poliovirus type 2 (cVDPV2) strains detected in wastewater samples, Finland, Germany, Poland, Spain, the United Kingdom, September–December 2024 (n = 101)

BCN: Barcelona, Spain; BON: Bonn, Germany; COL: Cologne, Germany; DRE: Dresden, Germany; DUS: Dusseldorf, Germany; EWR: East Worthing, the United Kingdom; HAM: Hamburg, Germany; LEE: Leeds Knostrop, the United Kingdom; LOB: London Beckton, the United Kingdom; LOC: London Crossness, the United Kingdom; MAI: Mainz, Germany; MUN: Munich, Germany; RZE: Rzeszow, Poland; TAM: Tampere, Finland; WAR: Warsaw, Poland.

As shown in Figure 2, VP1 sequence analysis revealed high sequence diversity between isolates from the same site or country but relatively high sequence identity between isolates from different countries, e.g. the two isolates from Poland are closer to other European isolates than they are between themselves; five of six isolates from the UK are closer to other European strains than they are to other UK isolates; and some isolates from Germany are closer to other European strains than they are to other German isolates. This observed genetic diversity argues for nearly simultaneous importations of genetically related viruses having occurred into different European locations from an unknown country or area during a short period of time although other explanations are possible, such as some transmission occurring between European countries.

The tree was drawn using Microreact software [9]. Labelling of strains include the city name and date of sample collection.

Given the frequency and extent of cVDPV2 detections, particularly in Germany, it cannot be ruled out that there is some level of poliovirus transmission following the importation event/s in Europe. However, this is a too short period of time for a sufficient number of mutations to have accumulated in sequential isolates to provide clear evidence and an estimate of the extent of virus transmission.

### Whole genome sequence analysis of circulating vaccine-derived poliovirus type 2 (cVDPV2) strains

**Figure 3:**
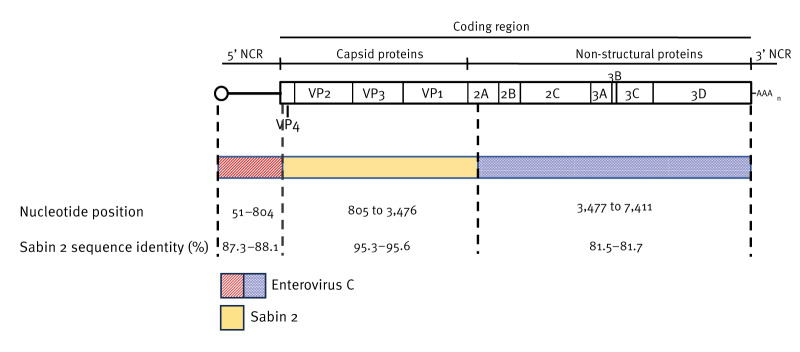
Schematic representation of circulating vaccine-derived poliovirus type 2 (cVDPV2) strain NIE-ZAS-1 double recombinant structure with sequences derived from Sabin 2 vaccine strain and unidentified enterovirus C strains, Europe, 2024 (n = 16)^a^

^a^ A total of 16 isolates were sequenced: Finland (n = 7), Germany (n = 4), Poland (n = 1) and the United Kingdom (n = 4).

Whole genome sequence analysis of selected European NIE-ZAS-1 isolates (n = 16) revealed a double recombinant structure with viruses having replaced sequences upstream and downstream the capsid coding region with those from unidentified enterovirus C strains (Figure 3). Given the extent of transmission of NIE-ZAS-1 cVDPV2 since 2020, this is not unexpected as polioviruses are known to frequently recombine with enterovirus C strains during their evolution in humans [12]. Consequently, the European cVDPV2 NIE-ZAS-1 strains are expected to have lost the attenuation properties of Sabin 2 and increased their potential to cause paralytical disease by replacing the 5’ non-coding region where the main attenuation mutation locates and, in addition, mutating at the secondary attenuation site in residue 143 of the VP1 capsid protein.

The range of sequence identity in the different genome segments between the sequenced isolates and Sabin 2 reference strain is shown. Approximate crossover positions are shown but they are imprecise due to multiple single mutations likely acquired after the recombination event.

## Discussion

The GPEI is based on three pillars: vaccination, surveillance and containment. Besides clinical systems like acute flaccid paralysis (AFP) surveillance, enterovirus or laboratory-based surveillance systems, testing of wastewater samples, if done systematically, may detect silent circulation of polioviruses before symptomatic cases occur [13]. The detection of cVDPV2 in five European countries described here highlights the usefulness of wastewater surveillance for poliovirus, particularly in polio-free countries using inactivated polio vaccine. Due to a low manifestation index and the possibility to be transmitted in vaccinated populations, polioviruses can circulate over a long period of time undetected and pose a risk for individuals who are not sufficiently protected by antibodies. Routine wastewater testing for poliovirus can inform public health authorities early so that adequate measures can be introduced in time. The affected countries included in this report have alerted national public health authorities about the results, undertaken public health risk assessments and put the information into the public domain [14-18]. Key to the risk assessment is a review of national, regional and local vaccination coverage data to identify communities with low vaccination coverage for targeted vaccination campaigns. It is unclear whether NIE-ZAS-1 cVDPV2 might have also been imported into other European countries. While some countries might be missing such events due to lack or limited wastewater surveillance, ES is conducted in at least 23 of the 53 countries within the WHO European Region so more detections might occur in the near future [19].

## Conclusions

The simultaneous detection of genetically related cVDPV2 strains in different sites in Europe further justifies keeping the PHEIC active, the importance of fully investigating any suspect AFP case and the necessity to continue conducting sensitive ES for poliovirus and increasing vaccination coverage to prevent symptomatic poliomyelitis cases.
